# Management of Enteric Methane Emissions in Ruminants Using Feed Additives: A Review

**DOI:** 10.3390/ani12243452

**Published:** 2022-12-07

**Authors:** Valiollah Palangi, Maximilian Lackner

**Affiliations:** 1 Department of Animal Science, Agricultural Faculty, Ataturk University, 25240 Erzurum, Turkey; 2 Department of Industrial Engineering, University of Applied Sciences Technikum Wien, Hoechstaedtplatz 6, 1200 Vienna, Austria; 3 Circe Biotechnologie GmbH, Kerpengasse 125, 1210 Vienna, Austria

**Keywords:** greenhouse gas, methane emission, ruminants, feed additives

## Abstract

**Simple Summary:**

Methane emission from enteric fermentation in ruminants is the single most relevant greenhouse gas source in agriculture, and it is amongst the largest anthropogenic ones. As ruminants are needed globally for meat, milk and other goods production on a huge scale, feed additives could offer an interesting solution to reduce CH_4_ emissions. Methane emission strategies are investigated to maintaining productivity and the overall health of the animal. Some strategies have shown to reduce the propagation and/or eliminate ruminal flora affecting the health and productivity of the animal. Therefore, identifying beneficial strategies leads to improving productivity and the health of the animal and environment.

**Abstract:**

In ruminants’ metabolism, a surplus of hydrogen is removed from the reduction reaction of NAD^+^ (nicotinamide adenine dinucleotide) by the formation of methane by methanogenic bacteria and archaea methanogens. The balance of calculations between VFA (volatile fatty acids), CO_2_, and CH_4_ indicates that acetate and butyrate play a role in methane production, while the formation of propionate maintains hydrogen and therefore reduces methane production. CH_4_ formation in ruminant livestock is not desired because it reduces feed efficiency and contributes to global warming. Therefore, numerous strategies have been investigated to mitigate methane production in ruminants. This review focuses on feed additives which have the capability of reducing methane emissions in ruminants. Due to the environmental importance of methane emissions, such studies are needed to make milk and meat production more sustainable. Additionally, the additives which have no adverse effects on rumen microbial population and where the reduction effects are a result of their hydrogen sink property, are the best reduction methods. Methane inhibitors have shown such a property in most cases. More work is needed to bring methane-reducing agents in ruminant diets to full market maturity, so that farmers can reap feed cost savings and simultaneously achieve environmental benefits.

## 1. Introduction

Grazing animals such as sheep, cattle and goats worldwide emit a huge amount of polluting gases, of which methane is first and foremost. An amount of approximately 86 million metric tons (Tg) of methane is produced by domesticated ruminants per year [1,2]. Saunois et al. [3] estimated total emissions of 111 (106–116) Tg CH_4_ yr^−1^ for enteric fermentation and manure management, about one-third of total global anthropogenic emissions (for the period 2008–2017).

**Figure 1:**
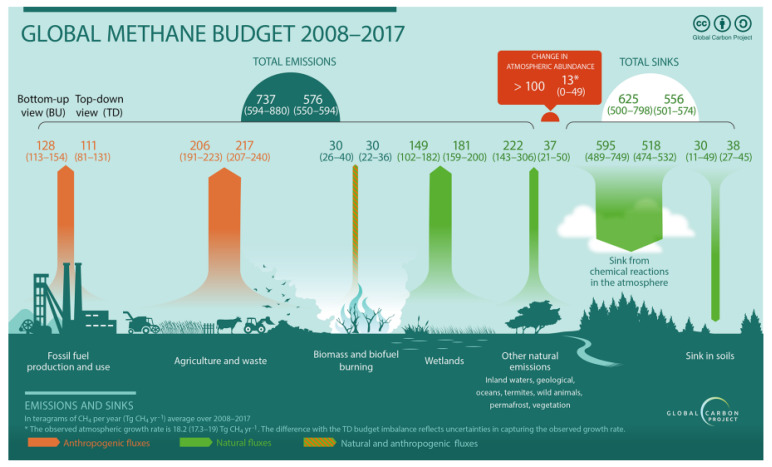
Methane flows on a global level per year for the period between 2008 and 2017. Numbers for sources and sinks are in Tg CH_4_ yr^−1^). Agriculture and waste are the largest anthropogenic sources with 191–240 Tg of CH_4_ per year. Reproduced with permission from Saunois et al. [3].

The emissions shown in Figure 1, for agriculture and waste amount to 191–240 Tg of CH_4_ per year, which translates into roughly 24–30 kg per capita, at a world population of 8 billion people. With a GWP (greenhouse warming potential) of 24 [4,5], that corresponds to 572–720 kg of CO_2_ (CO_2_ equivalents, CO_2e_) per person per year.

**Figure 2:**
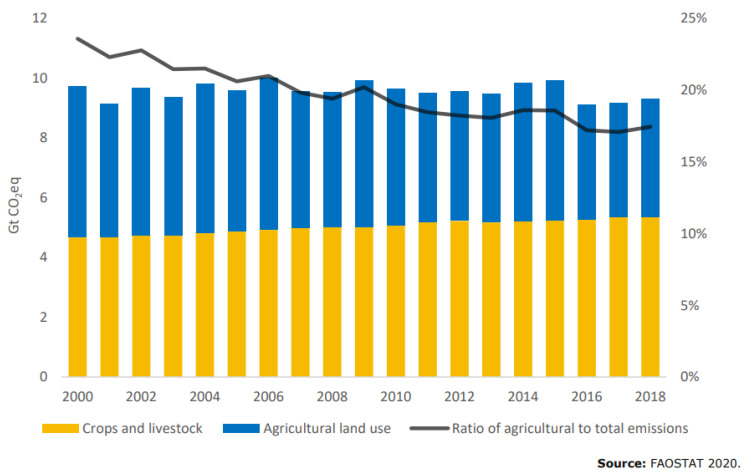
Yearly CO_2e_ emissions from crops and livestock (yellow) and agricultural land use (blue) and share of the sector in global GHG emissions (black). Reproduced with permission from FAO [6].

Figure 2, reproduced with permission from FAO [6], shows the trend in agricultural GHG (greenhouse gas) emissions where ruminants account for the single largest contribution with 39% globally within the sector. In some countries, that figure is even higher, e.g., in Australia at 70% [7].

In the metabolic pathway of ruminants, production of acetate and butyrate releases pure hydrogen, while propionate formation creates a competitive pathway for H+ use in the rumen [8]. Methane is a greenhouse gas that leads to energy loss in ruminants and plays a vital role in global warming [9]. Hence, actions to minimize enteric CH_4_ production and emission from ruminants not only limit the emission of GHG, but can also enhance production performance of the operations. Over the past few years, review papers and meta-analyses have been published on how different mitigation strategies influence CH_4_ production in ruminants, e.g., Eckard et al. [10], Cottle et al. [11], Hristov et al. [12], Broucek [13], Jeyanathan et al. [14], Yáñez-Ruiz et al. [15], McCauley et al. [16], Min et al. [17], Cardoso-Gutierrez et al. [18], and Palangi et al. [19]. Diet modification can be a good strategy for methane mitigation in ruminants. Granted, some additives may have adverse effects on the ruminal microbial flora. Additives such as nitrate and nitrite which have alternative hydrogen sink ability, might be the best for reduction methods. Furthermore, with the advent of genomic selection, including CH_4_ emissions as a breeding objective is attainable. However, in most cases, genetic selection has led to reduced rumen volume, which in reality has reduced the amount of fermentation.

However, given the importance of the topic and the fast pace of growing knowledge in the area, this article has tried to focus on bringing together and discussing the most recent findings, as well as feed additives that can be used as methane inhibitors in ruminants.

## 2. Use of Methane Inhibitors

Various methane inhibitors are added to the ration in order to prevent energy losses in the form of methane emission in ruminants, thus providing economic and ecological gains. One of these agents, bromomethane (CH_3_Br, CAS no. 74-83-9), was found to inhibit methane production by reacting with Coenzyme M, which is involved in the last step of methane formation [20]. Kim et al. [21] stated that 3-nitrooxypropanol (3NOP, HOCH_2_CH_2_CH_2_ONO_2_, CAS no. 100502-66-7) is a potential candidate as feed additive due to its methane mitigation effects, with no adverse effects on animal performance. Nitrate (NO_3_^−^), nitrite (NO_2_^−^) and 2-bromoethanesulfonic acid (C_2_H_5_BrSO_3_, CAS no. 26978-65-4) have similarly shown to decrease in vitro and in vivo methane production [22,23,24]. Nitrate and nitrite are alternative hydrogen sinks that draw hydrogen ions (H^+^) away from methanogenesis [25], while 2-bromoethanesulfonic acid inhibits the activity of methyl coenzyme M reductase [26]. Nitrate, propionic acid (CH_3_CH_2_COOH, CAS no. 79-09-4), 3-nitro-1-propionic acid (NO_2_CH_2_CH_2_COOH, CAS no. 7417-34-7), sulphate (SO_4_^2−^) and saponins (a group of secondary plant metabolites) have also been evaluated for their methanogenic inhibition effects alone and/or in combination showing promising results [27]. Some statins (HMG-CoA reductase inhibitors, a class of lipid-lowering medications) such as lovastatin (also called mevinolin), are formed by reduction of hydroxymethylglutaryl-SCoA (HMG-CoA). They have the potential to specifically inhibit methanogenic bacteria of the rumen [28]. Kim et al. [21] and Nkemka et al. [29], also observed a significant methane reduction, with 3-nitrooxypropanol (3NOP) supplementation. According to Rebelo et al. [30], the animals fed non-protein nitrogen had lower daily methane emission compared to soybean meal diets. In the study of Ramin et al. [31] with increasing levels of *Alaria esculenta* (seaweeds) fractions in the ration, methane production showed a linear decrease, indicating the inhibition of methane producing microbes by the treatments. Similarly, Alvarez-Hess et al. [32] noted that the addition of nitrate, fat and 3-nitrooxypropanol decreased in vitro methane production by 21, 19 and 44%, respectively. Natel et al. [33] demonstrated that the replacement of soybean meal by encapsulated nitrate products inhibited methane production via reducing the ruminal methanogens community.

## 3. Use of Herbal Extracts

In recent years, plant and herbal extracts have been successfully used as substitutes to antibiotics and feed additives in the livestock industry. Among plant-extracted material [34], essential oils (etheric oils) [35], saponins [36], tannins [37] and organosulfides [30] have shown promising results in improving rumen microbial population and nitrogen metabolism, reducing methane production and enhancing overall animal health and performance. According to Pérez-Barbería et al. [38], the ericaceae (calcifuges, plants that dislike alkaline (chalky)) soils, (e.g., heather, European blueberry, *Vaccinium myrtillus*) resulted in decreased methane emissions in red deer and sheep. Fandiño et al. [39] reported that the doses above 200 mg/d of anise (*Pimpinella anisum*, aniseed) and capsicum (*Chilli pepper*) oils mixture decreased the acetate to propionate ratio and increased the butyrate proportion while the doses above 375 mg/d increased dry matter intake. Hart et al. [40] noted the beneficial effects of essential oils on dairy cows’ performance, as well as reducing methane emission. In a study investigating the effects of thyme (*Thymus vulgaris*), mint (*Mentha piperita*) and orange (*Citrus sinensis*) oils on rumen fermentation, a significant decrease in methane and CO_2_ production was reported with increasing levels of essential oils added to the ration [41]. In agreement, *Santalum spicatum* essential oil treatment led to 50% methane production reduction [42]. Pedraza-Hernandez et al. [43] observed a decrease in methane and carbon dioxide emission from goats that were fed with the addition of *M. oleifera* (moringa) extract and *S. cerevisiae (Saccharomyces cerevisiae,* brewer’s yeast or baker’s yeast) in their diets. As previously discussed by Sinz et al. [44], the combination of certain plant extracts such as (acacia (*Acacia mearnsii*), grape (*Vitis vinifera* L.) seed and green tea (*Camellia sinensis*) extracts) led to a decrease in methane production. Furthermore, Wann et al. [45] noticed that inclusion of bamboo grass (*Tiliacora triandra*, Diels) pellets could lead to a reduction in methane production. The study by Abdelrahman et al. [46] investigated the influence of herbal extracts on methane production and reported that using eucalyptus (*Eucalyptus globulus*) oil could decrease methane production. Agarwal et al. [47] investigated the effects of mint oil on in vitro methanogenesis and fermentation parameters of buffalo rumen fluid. Roca-Fernández et al. [48] concluded that legumes containing condensed tannin concentrations also decreased methane production compared with the alfalfa diet. Inhibitory effects of some extracts and or essential oils may be due to their toxicity thus reducing rumen microorganism population, microbial fermentation and methanogenesis.

## 4. Use of Bee Propolis Extract

Propolis is a plant-origin bee product collected by honeybees from exudates and buds of various plant species. It can be utilized in animal nutrition as a dietary additive [49]. Propolis stimulates the rumen microorganisms for the consumption of hydrogen by changing in total volatile fatty acids (VFA), and it was suggested that there is a need to study the effect of propolis for the mitigation of methane-based emissions with regard to phytogeography, botanical origin, climatic conditions, and collection methods for further effective applications of propolis in the mitigation of methane in vivo [50]. Propolis phenolic compounds are known to cause the improvement of rumen fermentation, reduction of NH_3_-N [51] and methane emission [49]. Morsy et al. [52] illustrated that bee propolis extract possesses anti-methanogenic activity and reduces methane emission. Kara et al. [53] noted that propolis could reduce methane production in the rumen.

## 5. Use of Saponins

Saponins (AKA triterpene glycosides), as one of the biggest classes of phytochemicals, are found in many plants including *Yucca schidigera* and *Quillaja saponaria* which have been deployed as feed additives for years. These compounds not only are potential rumen modifiers but could also act as enteric methane production reducing material. A meta-analysis of the effects of saponin-rich sources on methane production and ruminal fermentation parameters examined through in vitro experiments found that adding saponin-rich sources not only reduced ruminal methane emission, but also reduced acetate proportion and increased propionate [54]. In vivo experiments on sheep showed that methane production was reduced as a result of adding *Sapindus saponaria* fruits [55] or *Yucca schidigera* [56]. However, other experiments on sheep reported no significant reduction in methane production compared to control groups by adding saponin-rich extracts of alfalfa (*Medicago sativa* L.) root [57], *Yucca schidigera* and *Quillaja saponaria* [58,59], and tea saponin [60]. Correspondingly, the addition of *Yucca schidigera* powder [61] and powder from the whole-plant *Quillaja saponaria* had insignificant effects on dairy cattle [62]. Similar results were reported by others where adding saponin-rich extracts of *Yucca schidigera* and *Quillaja Saponaria,* as well as tea saponin did not reduce CH_4_ production, and subsequently its ratio to DMI [58], tea saponin [63] and *Yucca schidigera* powder [64]. However, Mao et al. [65] found a significant reduction in CH_4_/DMI in the group that received tea saponin compared with the control group. It has been suggested that the effects of saponin in reducing CH_4_ are due to the reduction of protozoa (single-celled eukaryotes) or methanogenic archaea (a domain of single-celled organisms without cell nucleus = prokaryotes) counts [66]. In the same vein, a meta-analysis by Jayanegara et al. [54] reported a significant reduction in protozoa count at higher levels of saponin. Since dihydrogen (H_2_) is a key element involved in ruminal CH_4_ production, a lower number of protozoa, as hydrogen producers, can reduce CH_4_ production [67]. In other words, defaunation reduces the population of methanogens, resulting in lower CH_4_ production [54]. Wina et al. [68] suggested that a significant effect of saponin on acetate and propionate concentrations is how it changes these concentrations in a way that increased the corporation of propionate and decreased the acetate/propionate ratio. They also argued that this increase in propionate could be due to the lower levels of acetate and butyrate since those are among the main products of fermentation by protozoa. Correspondingly, saponin would lower protozoa count, thereby increasing propionate concentrations [68]. Tan et al. [69] concluded that different genera of rumen protozoa ciliates appear to be selectively inhibited by tea saponin. Saponins have shown potential as antiprotozoal agents to increase microbial supply to the host and decrease CH_4_ emissions [70]. This effect has been reported to be transitory due to the deglycosylation of saponins to sapogenins by rumen bacteria [71].

## 6. Use of Yeast

Recently, yeasts have found wide applications as an additive for ruminants to enhance their health, production performance and ruminal fermentation. A meta-analysis of 110 studies on the effects of *Saccharomyces cerevisiae* on ruminants indicated that yeast supplementation increased dry matter intake (DMI), milk yield, rumen pH, and VFA concentration while decreasing lactic acid concentration with no impact on the acetate-to-propionate ratio [72]. (*S. cerevisiae* is a species of yeast that is a single-celled fungal microorganism that has been instrumental in fermentation for making wine, beer and bread for several 1000 years). Relatively few in vivo experiments have been conducted thus far to examine the effects of (live or cultured) yeasts on CH_4_ production in ruminants. However, in vitro experiments demonstrated a positive effect of yeast culture and live yeast on mitigating CH_4_ production [73]. The investigation of the effect of live yeast on hydrogen consumption by two hydrogen-friendly bacteria in the rumen (one producing acetate and the other producing methane) showed that in the presence of yeast, acetogenic bacteria and their production of acetate increased up to five times. In the absence of yeast (in an environment where both of the above bacteria are present), hydrogen is primarily used to produce methane. Still, the presence of yeast stimulates the use of hydrogen by acetogenic bacteria and increases acetate production [74]. However, the degree of mitigation of CH_4_ output may vary from one experiment to another depending on the type of substrate, media components and yeast dosage. Results from in vivo experiments on effects of *S. cerevisiae* in CH_4_ production in dairy and beef cattle showed that yeast supplementation did not significantly affect the CH_4_ production and or CH_4_/DMI. In agreement, Muñoz et al. [75] and Bayat et al. [76] found no significant decrease in CH_4_ output or CH_4_/DMI in dairy cattle using active dry yeast supplementation at the dosage of 0.5 g per day. Studies on supplementation with yeast culture showed no mitigation in CH_4_ production and CH_4_/DMI [77]. In line with these findings, a meta-analysis also reported that yeast supplementation for the diets of dairy and beef cattle had no impact in terms of mitigating CH_4_ production [78], which can partially and/or completely be attributed to inappropriate dosage, yeast variety, and/or the duration of administration. By contrast, studies on the effects of yeast culture on CH_4_ production in sheep and goats demonstrated its positive impact in mitigating CH_4_ production. The addition of yeast culture to wethers’ (castrated male goats or sheep) diet at a concentration of 4 g/day resulted in a 10.19% and 6.85% reduction in CH_4_ production and CH_4_/DMI, respectively, compared with the control group [79]. The potency of yeast culture is dose-dependent since supplementation of 12 g/day to the diet of growing goats reduced CH_4_/DMI by 15% which is higher than the reported value by Mwenya et al. [79] and Lu et al. [80]. Dai et al. [81] meta-analyzed the effects of ruminal protozoa on CH_4_ emissions.

Although the exact mechanism through which yeast mitigates CH_4_ production is unknown, it has been suggested that reduced CH_4_ production due to yeast supplementation in rations is attributable to greater propionate production requiring the use of metabolic hydrogen and therefore reducing methanogenesis [82]. It has also been suggested by Shibata and Terada [83] that the molar proportions of VFAs are changed as a result of using probiotics (live microorganisms) for ruminants in a way that the ratio of acetate is decreased whereas that of propionate increases. However, the results reported by in vivo studies on ruminal fermentation patterns show that the same pattern does not hold for CH_4_ production and acetate and propionate concentrations. However, further experiments are needed to establish the effects of yeast in mitigating CH_4_ production in ruminants as responses may vary depending on yeast dosage, basal diets and forage/concentrate ratio. At this point, the reader is also referred to Palangi et al. [19].

## 7. Use of Ionophores

Ionophores are antibiotics. With a broad range of structures, they commonly have oxygen atoms that could create a cavity position for cations’ entrapment. Mc Guffey et al. [84] stated that these compounds bind to the membrane of the rumen microorganisms, thus changing the passage of cations passing through the membrane. Monensin (CAS no. 17090-79-8) is one of the ionophores that inhibits methanogens’ access to hydrogen ions by disrupting the transfer of hydrogen ions from the protozoa cell membrane. Guan et al. [85] reported that supplementation of ionophores to the ruminant diet was related to the transitory decline in ruminal ciliate protozoal populations. It can decrease the ruminal methane emission. Gupta et al. [86] indicates that monensin supplementation (about 0.6 mg/kg body weight (BW)) in growing heifers reduced enteric methane production (a heifer is a young cow before she has had her first calf).

## 8. Use of Organic Acids

Organic acids are used in farm animal rations for various purposes. Organic acids increase the acidity of the diet and prevent its deterioration (compare silage). The reduced pH improves the digestion and absorption of nutrients by maintaining the balance between pathogens and beneficial microorganisms in the digestive system [87]. Low doses of formic acid have significantly reduced the in vitro total gas production, yet at higher doses had an inverse effect on gas production Kara et al. [88]. Partanen and Jalava [89] stated that formic acid has a large inhibitory effect on total gas production. Palangi and Macit [9] reported that fumaric acid might be used sustainably by reducing the amount of methane emitted from ruminants and improving the environmental conditions.

## 9. Use of Exogenous Enzymes

Another additive used in the ruminant diet consists of enzymes with fibrolytic or proteolytic activities, which can improve plant cell walls’ digestibility, thereby enhancing production performance [90]. In vitro experiments on how using enzymes may mitigate CH_4_ production have produced mixed results. For example, adding cellulase led to a linear, quadratic decrease in CH_4_ production per unit of degraded DM [91], or supplementation with xylanase (enzymes that degrade the linear polysaccharide xylan into xylose, thus breaking down hemicellulose) increased CH_4_ in rice straw and grass substrates [92]. Contrarily, a mixture of cellulase (enzymes that decompose cellulose and related polysaccharides), xylanase, and beta-gluconase (glucanases are enzymes that break down large polysaccharides via hydrolysis). Beta-Glucanase hydrolyzes 1, 3 and 1, 4 glycosidic bonds as found in cereal endosperm cell walls and had no impact on CH_4_ production [93]. A handful of in vivo studies have been conducted to examine the effects of enzymes on CH_4_ production in ruminants. Proteolytic enzyme (enzymes which break down proteins) supplementation of beef cattle diets did not lead to any considerable influence on CH_4_ production, CH_4_/DMI, percent CH_4_ energy/GE intake (GE = gross energy), and ruminal fermentation pattern. However, the dry matter’s digestibility was enhanced by 8% compared with the control group [94]. Another notable finding concerning dairy cattle showed that CH_4_ production and CH_4_/DMI increased linearly with a higher dosage of fibrolytic enzyme (0, 0.5, 1 mL of enzyme/kg of TMR, total mixed ration, %DM), with no impact on methanogens or protozoa and bacteria communities or acetate, propionate and butyrate concentrations [95]. In other words, adding a high level of an enzyme to the diet resulted in a 16% increase in CH_4_ production (g/day), and 12% increase in CH_4_/DMI and a 16% increase in milk production. These findings suggest that a greater amount of energy was lost in the form of CH_4_ during ruminal fermentation [95]. Likewise, other studies reported no effects for cellulase and xylanase on CH_4_ production and ruminal methanogen community activity in growing goats [80], or exogenous enzymes derived from *Aspergillus oryzae* and *Aspergillus niger* on CH_4_ production or concentrations of ruminal acetate, propionate and butyrate in dairy cattle [77]. In contrast, by estimating CH_4_ through ruminal fermentation pattern in dairy cattle, Arriola et al. [96] reported that adding fibrolytic enzymes (enzymes that increase nutrient availability from cell walls) could potentially mitigate CH_4_ production and reduce acetate/propionate ratio. Although enzymes can improve the degradation of fibers and lessen the acetate/propionate ratio, further experiments are needed in this area as different observed responses could depend on the type of enzyme activity, dosage, diet composition and kind of substrate.

## 10. Use of Nanoparticles

Another effective strategy for enteric methane mitigation is functional nanoparticles with stronger absorption ability, and high specific surface area. Such materials have shown to increase the bioavailability of feeds. The ability of nanoparticles to penetrate cell membranes is the main feature of interaction with biological systems. In this way, interaction with the immune system, uptake, absorption, distribution, and metabolism is facilitated biologically [97]. The particle size conversion to a nanoscale (below 100 nanometers in at least one dimension, 1 nm = 10^−9^ m) increases the surface/volume ratio, and changes in other properties also occur. Increasing the contact surface in nanoparticles allows the interaction of such materials with different organic and inorganic molecules [98]. Moreover, Fujinawa et al. [99] showed that carbon nanoparticles specifically inhibit methanogens in an anaerobic environment.

Similarly, Jiang et al. [100] reported that granular activated carbon has an inhibitory effect against CH_4_ under anaerobic conditions.

On the one hand, Wang et al. [101] reported that magnesium oxide addition reduced the in vitro gas production volume and acetate molar percentage while increasing the propionate molar percentage. Magnesium oxide improves the rumen fermentation model by increasing the efficiency of microbial mass synthesis. Moreover, Kazemi and Vatandoost [102] demonstrated magnesium oxide increases organic matter degradability by decreasing the methane yield. On the other hand, zinc intake of the microbial population in ruminants causes changes in ruminal digestion and fermentation [103]. The use of zinc oxide nanoparticles in the ration increases in vitro rumen bacterial growth and increases energy intake efficiency [104]. Chanzanagh et al. [105] found that in the 24th hour of incubation, the total amount of in vitro gas production was the least in the group containing 60 ppm ZnO nanoparticles. Chen et al. [104] investigated the effect of different levels of nanoscale zinc oxide (nZnO) (at levels of 0, 50, 100, 200, and 400 mg/kg) on rumen fermentation, and the use of nanoparticles enabled the growth of rumen microorganisms and improved microbial protein synthesis and energy efficiency. In accordance, Maorong et al. [106] stated that copper supplementation increases rumen microorganisms’ growth and the concentration of essential fatty acids (EFA; EFA are polyunsaturated fatty acids (PUFA, omega-3 (ω-3) and omega-6 (ω-6)) that must be provided by foods because they cannot be synthesized by animals; however, they are needed.

Nonetheless, Hernández-Sánchez et al. [107] reported that the inclusion of different doses of elemental copper could reduce methane production.

## 11. Use of Algae

Micro- and macroalgae have been tested successfully as feedd additives, e.g., Anele et al. [108] and Brooke et al. [109], or the reviews by McCauley et al. [16] and Makkar et al. [110]. Machado et al. [111] first identified red algae reducing methanogenesis.

**Figure 3:**
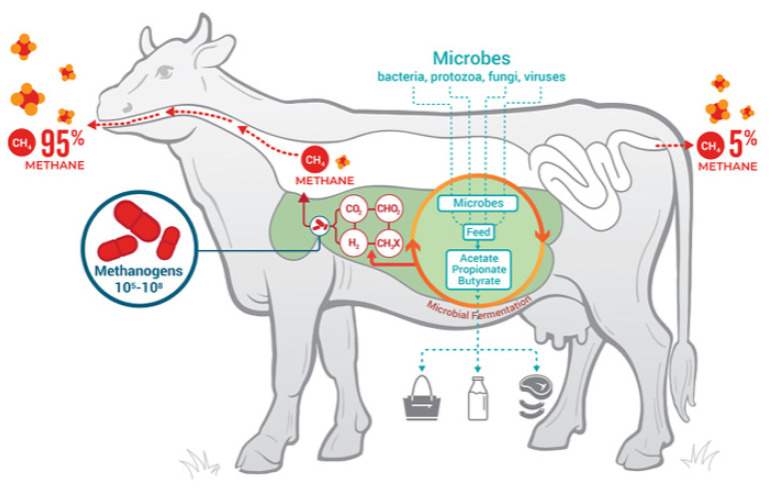
Ruminant fermentation processes, products, and microbial contributors. Glasson et al. [112].

As shown in Figure 3, most emissions of methane stem from eruction (95%); flatulence only accounts for 5%.

Bromoform (CHBr_3_) was found to be the strongest active compound in algae for the inhibition of methanogenesis. It needs to be understood what the effects are of that compound on the animal, and on atmospheric chemistry [112]. As stated by Min et al. [113], there are also some concerns as to the sustainable production of seaweeds, and their potential negative impacts on the rumen digestibility and health impacts of bromoform.

## 12. Discussion

Despite the huge potential to realize cuts in CH_4_ emissions from enteric fermentation, there has been little commercialization so far. An example of a successful commercial product is Bovaer™. The manufacturer states on its website: “A quarter teaspoon of Bovaer^®^ per cow per day suppresses the enzyme that triggers methane production in a cow’s rumen and consistently reduces enteric methane emission by approximately 30% for dairy cows and even higher percentages (up to 90%) for beef cows … in September 2021, DSM received its first full regulatory approval to commercialize Bovaer^®^ from the Brazilian and Chilean authorities, for application in beef, dairy, sheep and goats. In February 2022, DSM received EU market approval for Bovaer^®^ for dairy cows, following a positive EFSA Opinion which confirms that Bovaer^®^ reduces enteric methane emissions from dairy cows and is safe for the animal and the consumer. It is the first time a feed additive authorised in the EU for environmental benefits can be marketed” [114].

The active ingredient in Bovaer™ is 3NOP [115].

The Department of Primary Industries and Regional Development, Government of Western Australia [116] writes:

“There is potential for natural compounds and materials to reduce methane production in livestock, though these products have not been widely commercialised. Feeding one type of seaweed at 3% of the diet has resulted in up to 80% reduction in methane emissions from cattle.

Fats and oils show the most potential for practical application to farming systems and have shown methane emission reductions of 15–20%”.

It can be expected that the global meat and milk demand will continue to increase, triggered by a growing population and increased economic development and a concurrent surge in demand. To which extent, and over what time frame alternative protein products such as insect-based protein, single-cell protein (SCP), soy- and pea-based proteins, lab grown meat and other solutions will replace farm-grown meat from cattle, remains to be answered. In any case, one can assume a rising pressure on the livestock industry to reduce its climate impact, not only from land use change, but also from enteric fermentation where the largest lever resides. Given the high amount of CH_4_ emissions from enteric fermentation, particularly cattle but also other species, the commercialization of feed additives alongside farmer training, can be a very good approach to curb emissions. One can compare reduced enteric methane emissions to energy efficiency gains in other sectors. Energy which is not consumed is the cheapest and most effective way to avoid CO_2_ emissions. The same holds true for ruminants because CH_4_ that is not emitted in the first place is the best measure to combat climate change. It seems elusive to obtain a complete avoidance of enteric CH_4_ emissions, but even a small reduction on the order of 10–30% would have a strong impact. Based on the numbers provided above, 20–70 Tg of avoided CH_4_ emissions per year could be achieved, which equals ~0.5 to 1.7 billion tons of CO_2e_ per year. For an assessment on the mitigation potential for Australia, see Black et al. [117].

Despite the negative effects of ruminant livestock on the climate, Gill et al. report that the safeguarding of food security needs to be mentioned [118]. The alternative protein which is most likely to close a gap in protein supply in case of a sudden, global food/feed catastrophe is considered to be bacterial single cell protein [119].

It will be necessary to convince farmers of different herd sizes to adopt low methane strategies. Since lower methane emissions equates with higher feed efficiency and, hence, improved economics, it should be possible to get buy-in from the entire ruminant value chain. The economic gains are immediate, and the climate benefits are more mid- and long-term. One must not forget that climate change exerts a negative feedback loop in livestock production (e.g., Palangi et al. [19], and Lackner et al. [5]), so reducing methane emissions is in the best interests of those who produce meat and milk with ruminant animals. It is also possible to claim carbon credits for enteric methane reduction. For instance, in Australia there are currently two approved methodologies for using feed additives or supplements to reduce methane emissions according to the Department of Primary Industries and Regional Development, Government of Western Australia [116]:

- feeding nitrates (for beef cattle)
- feeding dietary additives (for milking cows)

In addition, carbon credits may be claimed from these measures (Department of Primary Industries and Regional Development, Government of Western Australia [116].

Therefore, there exists technical solutions to reduce ruminants’ CH_4_ emissions, and economic models that show how to benefit from such measures that have been developed.

In this review, we have summarized more than 40 different agents used in reducing enteric methane formation and emission. When choosing the feed additive, the following aspects, apart from effectiveness & efficiency, should be taken into account:

- possible toxicity to the ruminant
- potential environmental impacts/undesired side effects

These two aspects are related to clarity of use, as the risks are strongly associated with non-optimum deployment by the farmer where, for example, the ruminants consume a high dose of the additive.

For instance, essential oils and tannins were found to exert their methane-depressing effect due to toxicity. Antibiotics clearly can have detrimental effects on the environment, such as the formation of multi-resistant bacteria which can also harm humans, so care needs to be taken. The same holds true for non-natural compounds being administered, such as chemicals (e.g., bromomethane) and certain nanoparticles, as well as products that do not occur in the natural environment of the ruminants such as seaweeds.

The literature offers limited clues to whether the mentioned feed additives alter host digestive performance or metabolic function, affect gene expression of the host ruminants, or alter gut flora abundance, as the studies are typically limited to showing a principal effect observed in the lab, and not a full explanation of the mechanism. The technology readiness level (TRL) of most of the (promising) feed additives can hence be considered low, i.e., too low for large scale deployment.

The safest way for a farmer who aims to reduce feed losses and the environmental impact of their herd is the use of a commercial, i.e., fully tested, verified, and approved product, with clear dosing and application rules. Unfortunately, the offers available on the market are limited.

The authors encourage further tests and deployment to reduce the sectors’ GHG emissions in an effective and efficient way.

## 13. Conclusions

Ruminant-emitted methane greatly contributes to greenhouse gas (GHG) emissions, thus strategies are being developed and investigated to mitigate methane production while maintaining productivity and the overall health of the animals. Some strategies have been shown to reduce the propagation and/or eliminate ruminal flora affecting the health and productivity of the animal. Therefore, summarizing these strategies as well as presenting their strengths and weaknesses can pave the way for further and purposeful research. In conclusion, all of the strategies mentioned above have the potential to efficiently and effectively reduce methane production; however, the question of the “best” approach has not yet been answered. This remains a serious challenge requiring further research and attention, and a need for several strategies, which may emerge depending on geographic region and other factors.

## Data Availability

All data generated during the study are included in the published article(s) cited within the text and acknowledged in the reference section.

## References

[1] Hook S.E., Wright A.D.G., McBride B.W. (2010). Methanogens: Methane producers of the rumen and mitigation strategies. Archaea.

[2] Ghanbari Maman L., Palizban F., Fallah Atanaki F., Elmi Ghiasi N., Ariaeenejad S., Ghaffari M.R., Kavousi K. (2020). Co-abundance analysis reveals hidden players associated with high methane yield phenotype in sheep rumen microbiome. Sci. Rep..

[3] Saunois M., Stavert A.R., Poulter B., Bousquet P., Canadell J.G., Jackson R.B., Zhuang Q. (2020). The global methane budget 2000–2017. Earth Sys. Sci. Data.

[4] EPA, Overview of Greenhouse Gasses. https://www.epa.gov/ghgemissions/overview-greenhouse-gases,.

[5] Lackner M., Sajjadi B., Chen W. (2022). Handbook of Climate Change Mitigation and Adaptation.

[6] FAO (2018). FAO Stat Analytical Brief 18, Emissions Due to Agriculture—Global, Regional and Country Trends 2000–2018. https://www.fao.org/3/cb3808en/cb3808en.pdf.

[7] (2022). Department of Primary Industries and Regional Development, Government of Western Australia. https://www.agric.wa.gov.au/climate-change/carbon-farming-reducing-methane-emissions-cattle-using-feed-additives.

[8] Palangi V., Macit M., Nadaroglu H., Taghizadeh A. (2022). Effects of green-synthesized CuO and ZnO nanoparticles on ruminal mitigation of methane emission to the enhancement of the cleaner environment. Biomass Convers. Biorefinery.

[9] Palangi V., Macit M. (2021). Indictable mitigation of methane emission using some organic acids as additives towards a cleaner ecosystem. Waste Biomass Valorization.

[10] Eckard R.J., Grainger C., de Klein C.A.M. (2010). Options for the abatement of methane and nitrous oxide from ruminant, production: A review. Livest. Sci..

[11] Cottle D.J., Nolan J.V., Wiedemann S.G. (2011). Ruminant enteric methane mitigation: A review. Anim. Prod. Sci..

[12] Hristov A.N., Oh J., Firkins J.L., Dijkstra J., Kebreab E., Waghorn G., Tricarico J.M. (2013). Special topics—Mitigation of methane and nitrous oxide emissions from animal operations: I. A review of enteric methane mitigation options. J. Anim. Sci..

[13] Broucek J. (2014). Production of Methane Emissions from Ruminant Husbandry: A Review. J. Environ. Prot..

[14] Jeyanathan J., Martin C., Morgavi D.P. (2014). The use of direct-fed microbials for mitigation of ruminant methane emissions: A review. Animal.

[15] Yáñez-Ruiz D.R., Bannink A., Dijkstra J., Kebreab E., Morgavi D.P., O’Kiely P., Hristov A.N. (2016). Design, implementation and interpretation of in vitro batch culture experiments to assess enteric methane mitigation in ruminants—A review. Anim. Feed Sci. Technol..

[16] McCauley J.I., Labeeuw L., Jaramillo-Madrid A.C., Nguyen L.N., Nghiem L.D., Chaves A.V., Ralph P.J. (2020). Management of enteric methanogenesis in ruminants by algal-derived feed additives. Curr. Pollut. Rep..

[17] Min B.R., Solaiman S., Waldrip H.M., Parker D., Todd R.W., Brauer D. (2020). Dietary mitigation of enteric methane emissions from ruminants: A review of plant tannin mitigation options. Anim. Nut..

[18] Cardoso-Gutierrez E., Aranda-Aguirre E., Robles-Jimenez L.E., Castelán-Ortega O.A., Chay-Canul A.J., Foggi G., González-Ronquillo M. (2021). Effect of tannins from tropical plants on methane production from ruminants: A systematic review. Vet. Anim. Sci..

[19] Palangi V., Taghizadeh A., Abachi S., Lackner M. (2022). Strategies to mitigate enteric methane emissions in ruminants: A review. Sustainability.

[20] Zhenming Z., Meng Q., Yu Z. (2011). Effects of methanogenic inhibitors on methane production and abundances of methanogens and cellulolytic bacteria in in vitro ruminal cultures. Appl. Environ. Microbiol..

[21] Kim H., Lee H.G., Baek Y.C., Lee S., Seo J. (2020). The effects of dietary supplementation with 3-nitrooxypropanol on enteric methane emissions, rumen fermentation, and production performance in ruminants: A meta-analysis. J. Anim. Sci. Technol..

[22] Patra A.K., Yu Z. (2014). Combinations of nitrate, saponin, and sulfate additively reduce methane production by rumen cultures in vitro while not adversely affecting feed digestion, fermentation or microbial communities. Bioresour. Technol..

[23] Newbold J.R., Van Zijderveld S.M., Hulshof R.B.A., Fokkink W.B., Leng R.A., Terencio P., Perdok H.B. (2014). The effect of incremental levels of dietary nitrate on methane emissions in Holstein steers and performance in Nelore bulls. J. Anim. Sci..

[24] Troy S.M., Duthie C.A., Hyslop J.J., Roehe R., Ross D.W., Wallace R.J., Rooke J.A. (2015). Effectiveness of nitrate addition and increased oil content as methane mitigation strategies for beef cattle fed two contrasting basal diets. J. Anim. Sci..

[25] Yang C., Rooke J.A., Cabeza I., Wallace R.J. (2016). Nitrate and inhibition of ruminal methanogenesis: Microbial ecology, obstacles, and opportunities for lowering methane emissions from ruminant livestock. Front. Microbiol..

[26] Balch W.E., Fox G.E., Magrum L.J., Woese C.R., Wolfe R.S. (1979). Methanogens: Reevaluation of a unique biological group. Microbiol. Rev..

[27] Wu H., Meng Q., Zhou Z., Yu Z. (2019). Ferric citrate, nitrate, saponin and their combinations affect in vitro ruminal fermentation, production of sulphide and methane and abundance of select microbial populations. J. Appl. Microbiol..

[28] Miller T.L., Wolin M.J. (2001). Inhibition of growth of methane-producing bacteria of the ruminant forestomach by hydroxymethylglutaryl∼SCoA reductase inhibitors. J. Dairy Sci..

[29] Nkemka V.N., Beauchemin K.A., Hao X. (2019). Treatment of feces from beef cattle fed the enteric methane inhibitor 3-nitrooxypropanol. Water. Sci. Technol..

[30] Rebelo L.R., Luna I.C., Messana J.D., Araujo R.C., Simioni T.A., Granja-Salcedo Y.T., Vitoa E.S., Lee C., Teixeira I.A.M.A., Rooke J.A. (2019). Effect of replacing soybean meal with urea or encapsulated nitrate with or without elemental sulfur on nitrogen digestion and methane emissions in feedlot cattle. Anim. Feed Sci. Technol..

[31] Ramin M., Franco M., Roleda M.Y., Aasen I.M., Hetta M., Steinshamn H. (2019). In vitro evaluation of utilisable crude protein and methane production for a diet in which grass silage was replaced by different levels and fractions of extracted seaweed proteins. Anim. Feed Sci. Technol..

[32] Alvarez-Hess P.S., Moate P.J., Williams S.R.O., Jacobs J.L., Beauchemin K.A., Hannah M.C., Durmic Z., Eckard R.J. (2019). Effect of combining wheat grain with nitrate, fat or 3-nitrooxypropanol on in vitro methane production. Anim. Feed Sci. Technol..

[33] Natel A.S., Abdalla A.L., Araujo R.C., McManus C., Paim T.P., Filho A.L.A., Louvandini P., Nazato C. (2019). Encapsulated nitrate replacing soybean meal changes in vitro ruminal fermentation and methane production in diets differing in concentrate to forage ratio. Anim. Sci. J..

[34] Ugbogu E.A., Elghandour M.M., Ikpeazu V.O., Buendía G.R., Molina O.M., Arunsi U.O., Salem A.Z. (2019). The potential impacts of dietary plant natural products on the sustainable mitigation of methane emission from livestock farming. J. Clean. Prod..

[35] Zhou R., Wu J., Lang X., Liu L., Casper D.P., Wang C., Zhang L., Wei S. (2020). Effects of oregano essential oil on in vitro ruminal fermentation, methane production, and ruminal microbial community. J. Dairy Sci..

[36] Adegbeye M.J., Elghandour M.M., Monroy J.C., Abegunde T.O., Salem A.Z., Barbabosa-Pliego A., Faniy T.O. (2019). Potential influence of Yucca extract as feed additive on greenhouse gases emission for a cleaner livestock and aquaculture farming—A review. J. Clean. Prod..

[37] Patra A.K., Min B.R., Saxena J. (2012). Dietary tannins on microbial ecology of the gastrointestinal tract in ruminants. Dietary Phytochemicals and Microbes.

[38] Pérez-Barbería F.J., Mayes R.W., Giráldez J., Sánchez-Pérez D. (2020). Ericaceous species reduce methane emissions in sheep and red deer: Respiration chamber measurements and predictions at the scale of European heathlands. Sci. Total Environ..

[39] Fandiño I., Ferret A., Calsamiglia S. (2020). Dose and combinations of anise oil and capsicum oleoresin as rumen fermentation modifiers in vitro and in vivo with high concentrate diets fed to Holstein beef heifers. Anim. Feed Sci. Technol..

[40] Hart K.J., Jones H.G., Waddams K.E., Worgan H.J., Zweifel B., Newbold C.J. (2019). An essential oil blend decreases methane emissions and increases milk yield in dairy cows. Open J. Anim. Sci..

[41] Canbolat Ö., Kalkan H., Karaman Ș., Filya I. (2011). The effect of essential oils on the digestibility, rumen fermentation and microbial protein production. Kafkas Üniv. Vet. Fak. Der..

[42] Jahani-Azizabadi H., Durmic Z., Vadhanabhuti J., Vercoe P.E. (2019). Effect of some australian native shrubs essential oils on in vitro rumen microbial fermentation of a high-concentrate diet. J. Anim. Plant Sci..

[43] Pedraza-Hernandez J., Elghandour M.M.M.Y., Khusro A., Camacho-Diaz L.M., Vallejo L.H., Barbabosa-Pliego A., Salem A.Z.M. (2019). Mitigation of ruminal gases production from goats using Moringa oleifera extract and live yeast culture for a cleaner agriculture environment. J. Clean. Prod..

[44] Sinz S., Marquardt S., Soliva C.R., Braun U., Liesegang A., Kreuzer M. (2019). Phenolic plant extracts are additive in their effects against in vitro ruminal methane and ammonia formation. Asian-Australas. J. Anim. Sci..

[45] Wann C., Wanapat M., Mapato C., Ampapon T., Huang B. (2019). Effect of bamboo grass (*Tiliacora triandra*, Diels) pellet supplementation on rumen fermentation characteristics and methane production in Thai native beef cattle. Asian-Australas. J. Anim. Sci..

[46] Abdelrahman S.M., Li R.H., Elnahr M., Farouk M.H., Lou Y. (2019). Effects of different levels of eucalyptus oil on methane production under in vitro conditions. Pol. J. Environ. Stud..

[47] Agarwal N., Shekhar C., Kumar R., Chaudhary L.C., Kamra D.N. (2009). Effect of peppermint (*Mentha piperita*) oil on in vitro methanogenesis and fermentation of feed with buffalo rumen liquor. Anim. Feed Sci. Technol..

[48] Roca-Fernández A.I., Dillard S.L., Soder K.J. (2020). Ruminal fermentation and enteric methane production of legumes containing condensed tannins fed in continuous culture. J. Dairy Sci..

[49] Santos N.W., Zeoula L.M., Yoshimura E.H., Machado E., Macheboeuf D., Cornu A. (2016). Brazilian propolis extract used as an additive to decrease methane emissions from the rumen microbial population in vitro. Trop. Anim. Health Prod..

[50] Morsy A.S., Soltan Y.A., Sallam S.M.A., Kreuzer M., Alencar S.M., Abdalla A.L. (2015). Comparison of the in vitro efficiency of supplementary bee propolis extracts of different origin in enhancing the ruminal degradability of organic matter and mitigating the formation of methane. Anim. Feed Sci. Technol..

[51] Ehtesham S., Vakili A.R., Danesh Mesgaran M., Bankova V. (2018). The effects of phenolic compounds in Iranian propolis extracts on in vitro rumen fermentation, methane production and microbial population. Iranian J. Appl. Anim. Sci..

[52] Morsy A.S., Soltan Y.A., El-Zaiat H.M., Alencar S.M., Abdalla A.L. (2021). Bee propolis extract as a phytogenic feed additive to enhance diet digestibility, rumen microbial biosynthesis, mitigating methane formation and health status of late pregnant ewes. Anim. Feed Sci. Technol..

[53] Kara K., Güçlü B.K., Oğuz F.K. (2014). Use of propolis and phenolic acids in ruminant nutrition. Erciyes Üniv. Vet. Fak. Der..

[54] Jayanegara A., Wina E., Takahashi J. (2014). Meta-analysis on methane mitigating properties of saponin-rich sources in the rumen: Influence of addition levels and plant sources. Asian-Australas. J. Anim. Sci..

[55] Hess H.D., Beuret R.A., Lotscher M., Hindrichsen I.K., Machmuller A., Carulla J.E., Kreuzer M. (2004). Ruminal fermentation, methanogenesis and nitrogen utilization of sheep receiving tropical grass hay-concentrate diets offered with Sapindus saponaria fruits and Cratylia argentea foliage. Anim. Sci..

[56] Wang C.J., Wang S.P., Zhou H. (2009). Influences of flavomycin, ropadiar, and saponin on nutrient digestibility, rumen fermentation, and methane emission from sheep. Anim. Feed Sci. Technol..

[57] Klita P.T., Mathison G.W., Fenton T.W., Hardin R.T. (1996). Effects of alfalfa root saponins on digestive function in sheep. J. Anim. Sci..

[58] Pen B., Takaura K., Yamaguchi S., Asa R., Takahashi J. (2007). Effects of *Yucca schidigera* and *Quillaja saponaria* with or without β 1–4 galacto-oligosaccharides on ruminal fermentation, methane production and nitrogen utilization in sheep. Anim. Feed Sci. Technol..

[59] Śliwiński B.J., Kreuzer M., Wettstein H.R., Machmüller A. (2002). Rumen fermentation and nitrogen balance of lambs fed diets containing plant extracts rich in tannins and saponins, and associated emissions of nitrogen and methane. Arch. Anim. Nutr..

[60] Liu Y., Ma T., Chen D., Zhang N., Si B., Deng K., Diao Q. (2019). Effects of tea saponin supplementation on nutrient digestibility, methanogenesis, and ruminal microbial flora in Dorper crossbred ewe. Animals.

[61] Van Zijderveld S.M., Dijkstra J., Perdok H.B., Newbold J.R., Gerrits W.J.J. (2011). Dietary inclusion of diallyl disulfide, yucca powder, calcium fumarate, an extruded linseed product, or medium-chain fatty acids does not affect methane production in lactating dairy cows. J. Dairy Sci..

[62] Holtshausen L., Chaves A.V., Beauchemin K.A., McGinn S.M., McAllister T.A., Odongo N.E., Benchaar C. (2009). Feeding saponin-containing *Yucca schidigera* and *Quillaja saponaria* to decrease enteric methane production in dairy cows. J. Dairy Sci..

[63] Yuan Z.P., Zhang C.M., Zhou L., Zou C.X., Guo Y.Q., Li W.T., Wu Y.M. (2007). Inhibition of methanogenesis by tea saponin and tea saponin plus disodium fumarate in sheep. J. Anim. Feed Sci..

[64] Santoso B., Mwenya B., Sar C., Gamo Y., Kobayashi T., Morikawa R., Takahashi J. (2004). Effects of supplementing galacto-oligosaccharides, *Yucca schidigera* or nisin on rumen methanogenesis, nitrogen and energy metabolism in sheep. Livest. Prod. Sci..

[65] Mao H.L., Wang J.K., Zhou Y.Y., Liu J.X. (2010). Effects of addition of tea saponins and soybean oil on methane production, fermentation and microbial population in the rumen of growing lambs. Livest. Sci..

[66] Patra A.K., Saxena J. (2009). The effect and mode of action of saponins on the microbial populations and fermentation in the rumen and ruminant production. Nut. Res. Rev..

[67] Morgavi D.P., Eugène M., Martin C., Doreau M. (2011). Reducing methane emissions in ruminants: Is it an achievable goal. Challenging Strategies to Promote the Sheep and Goat Sector in the Current Global Context. Options Méditerranéennes: Série A. Séminaires Méditerranéens.

[68] Wina E., Muetzel S., Hoffmann E., Makkar H.P.S., Becker K. (2005). Saponins containing methanol extract of *Sapindus rarak* affect microbial fermentation, microbial activity and microbial community structure in vitro. Anim. Feed Sci. Technol..

[69] Tan C., Ramírez-Restrepo C.A., Shah A.M., Hu R., Bell M., Wang Z., McSweeney C. (2020). The community structure and microbial linkage of rumen protozoa and methanogens in response to the addition of tea seed saponins in the diet of beef cattle. Anim. Feed Sci. Technol..

[70] Newbold C.J., De La Fuente G., Belanche A., Ramos-Morales E., McEwan N.R. (2015). The role of ciliate protozoa in the rumen. Front. Microbiol..

[71] Wallace R.J., McEwan N.R., McIntosh F.M., Teferedegne B., Newbold C.J. (2002). Natural products as manipulators of rumen fermentation. Asian-Australas. J. Anim. Sci..

[72] Desnoyers M., Giger-Reverdin S., Bertin G., Duvaux-Ponter C., Sauvant D. (2009). Meta-analysis of the influence *of Saccharomyces cerevisiae* supplementation on ruminal parameters and milk production of ruminants. J. Dairy Sci..

[73] O’brien M., Navarro-Villa A., Purcell P.J., Boland T.M., O’Kiely P. (2014). Reducing in vitro rumen methanogenesis for two contrasting diets using a series of inclusion rates of different additives. Anim. Prod. Sci..

[74] Chaucheyras F., Fonty G., Bertin G., Gouet P. (1995). In vitro H_2_ utilization by a ruminal acetogenic bacterium cultivated alone or in association with an Archaea methanogen is stimulated by a probiotic strain of Saccharomyces cerevisiae. Appl. Environ. Microbiol..

[75] Muñoz C., Wills D.A., Yan T. (2017). Effects of dietary active dried yeast (*Saccharomyces cerevisiae*) supply at two levels of concentrate on energy and nitrogen utilisation and methane emissions of lactating dairy cows. Anim. Prod. Sci..

[76] Bayat A.R., Kairenius P., Stefański T., Leskinen H., Comtet-Marre S., Forano E., Shingfield K.J. (2015). Effect of camelina oil or live yeasts (*Saccharomyces cerevisiae*) on ruminal methane production, rumen fermentation, and milk fatty acid composition in lactating cows fed grass silage diets. J. Dairy Sci..

[77] Oh J., Harper M., Melgar A., Compart D.P., Hristov A.N. (2019). Effects of *Saccharomyces cerevisiae*-based direct-fed microbial and exogenous enzyme products on enteric methane emission and productivity in lactating dairy cows. J. Dairy Sci..

[78] Darabighane B., Salem A.Z.M., Aghjehgheshlagh F.M., Mahdavi A., Zarei A., Elghandour M.M.M.Y., López S. (2019). Environmental efficiency of *Saccharomyces cerevisiae* on methane production in dairy and beef cattle via a meta-analysis. Environ. Sci. Pollut..

[79] Mwenya B., Santoso B., Sar C., Gamo Y., Kobayashi T., Arai I., Takahashi J. (2004). Effects of including β1–4 galacto-oligosaccharides, lactic acid bacteria or yeast culture on methanogenesis as well as energy and nitrogen metabolism in sheep. Anim. Feed Sci. Technol..

[80] Lu Q., Wu J., Wang M., Zhou C., Han X., Odongo E.N., Tang S. (2016). Effects of dietary addition of cellulase and a *Saccharomyces cerevisiae* fermentation product on nutrient digestibility, rumen fermentation and enteric methane emissions in growing goats. Arch. Anim. Nut..

[81] Dai X., Kalscheur K.F., Huhtanen P., Faciola A.P. (2022). Effects of ruminal protozoa on methane emissions in ruminants—A meta-analysis. J. Dairy Sci..

[82] Mutsvangwa T., Edwards I.E., Topps J.H., Paterson G.F.M. (1992). The effect of dietary inclusion of yeast culture (Yea-Sacc) on patterns of rumen fermentation, food intake and growth of intensively fed bulls. Anim. Sci..

[83] Shibata M., Terada F. (2010). Factors affecting methane production and mitigation in ruminants. Anim. Sci. J..

[84] McGuffey R.K., Richardson L.F., Wilkinson J.I.D. (2001). Ionophores for dairy cattle: Current status and future outlook. J. Dairy Sci..

[85] Guan H., Wittenberg K.M., Ominski K.H., Krause D.O. (2006). Efficacy of ionophores in cattle diets for mitigation of enteric methane. J. Anim. Sci..

[86] Gupta S., Mohini M., Malla B.A., Mondal G., Pandita S. (2019). Effects of monensin feeding on performance, nutrient utilization and enteric methane production in growing buffalo heifers. Trop. Anim. Health Pro..

[87] Palangi V. (2019). Effects of Processing Legume Forages with Organic Acids on In Vitro Gas Production, Rumen Fermantation and Methan Production. Ph.D. Thesis.

[88] Kara K., Aktuğ E., Çağrı A., Güçlü B.K., Baytok E. (2015). Effect of formic acid on in vitro ruminal fermentation and methane emission. Turkish J. Agric. Food Sci. Technol..

[89] Partanen K., Jalava T. (2005). Effects of some organic acids and salts on microbial fermentation in the digestive tract of piglets estimated using an in vitro gas production technique. Agric. Food Sci..

[90] Meale S.J., Beauchemin K.A., Hristov A.N., Chaves A.V., McAllister T.A. (2014). Board-invited review: Opportunities and challenges in using exogenous enzymes to improve ruminant production. J. Anim. Sci..

[91] Tang S.X., Zou Y., Wang M., Salem A.Z.M., Odongo N.E., Zhou C.S., Kang J.H. (2013). Effects of Exogenous Cellulase Source on In Vitro Fermentation Characteristics and Methane Production of Crop Straws and Grasses. Anim. Nutr. Feed Technol..

[92] He Z.X., Yang L.Y., Yang W.Z., Beauchemin K.A., Tang S.X., Huang J.Y., Tan Z.L. (2015). Efficacy of exogenous xylanases for improving in vitro fermentation of forages. J. Agric. Sci..

[93] Mohamed M.A.E., Yangchun C., Bodinga B.M., Lixin Z., Zekun Y., Lihui L., Wen L. (2017). Research article effect of exogenous fibrolytic enzymes on ruminal fermentation and gas production by RUSITEC, in vitro Abomasum and Ileum digestibility. Int. J. Pharmacol..

[94] McGinn S.M., Beauchemin K.A., Coates T., Colombatto D. (2004). Methane emissions from beef cattle: Effects of monensin, sunflower oil, enzymes, yeast, and fumaric acid. J. Anim. Sci..

[95] Chung Y.H., Zhou M., Holtshausen L., Alexander T.W., McAllister T.A., Guan L.L., Beauchemin K.A. (2012). A fibrolytic enzyme additive for lactating Holstein cow diets: Ruminal fermentation, rumen microbial populations, and enteric methane emissions. J. Dairy Sci..

[96] Arriola K.G., Kim S.C., Staples C.R., Adesogan A.T. (2011). Effect of fibrolytic enzyme application to low-and high-concentrate diets on the performance of lactating dairy cattle. J. Dairy Sci..

[97] Abdelsalam E., Samer M., Attia Y.A., Abdel-Hadi M.A., Hassan H.E., Badr Y. (2016). Comparison of nanoparticles effects on gas and methane production from anaerobic digestion of cattle dung slurry. Renew. Energy.

[98] Hernández-Sierra J.F., Ruiz F., Pena D.C.C., Martínez-Gutiérrez F., Martínez A.E., Guillén A.D.J.P., Castañón G.M. (2008). The antimicrobial sensitivity of *Streptococcus mutans* to nanoparticles of silver, zinc oxide, and gold. Nanomed. Nanotechnol. Biol. Med..

[99] Fujinawa K., Nagoya M., Kouzuma A., Watanabe K. (2019). Conductive carbon nanoparticles inhibit methanogens and stabilize hydrogen production in microbial electrolysis cells. Appl. Microbiol. Biotechnol..

[100] Jiang Q., Liu H., Zhang Y., Cui M.H., Fu B., Liu H.B. (2021). Insight into sludge anaerobic digestion with granular activated carbon addition: Methanogenic acceleration and methane reduction relief. Bioresour. Technol..

[101] Wang R., Si H.B., Wang M., Lin B., Deng J.P., Tan L.W., Tan Z.L. (2019). Effects of elemental magnesium and magnesium oxide on hydrogen, methane and volatile fatty acids production in in vitro rumen batch cultures. Anim. Feed Sci. Technol..

[102] Kazemi M., Vatandoost M. (2019). The effect of different levels of magnesium oxide with high purity on digestion-fermentation characteristics and methane emissions of a high-concentrate diet in the in vitro batch culture. J. Anim. Environ..

[103] Salem A.Z.M., Ammar H., Lopez S., Gohar Y.M., González J.S. (2011). Sensitivity of ruminal bacteria isolates of sheep, cattle and buffalo to some heavy metals. Anim. Feed Sci. Technol..

[104] Chen J., Wang W., Wang Z. (2011). Effect of nano-zinc oxide supplementation on rumen fermentation in vitro. Chin. J. Anim. Nutr..

[105] Chanzanagh E.G., Seifdavati J., Gheshlagh F.M.A., Benamar H.A., Sharifi R.S. (2018). Effect of ZnO nanoparticles on in vitro gas production of some animal and plant protein sources. Kafkas Üniv. Vet. Fak. Der..

[106] Maorong W., Fang M., Wenbin Y., Yingxiang H., Chaohua M., Feng W., Yao C. (2008). Influence of copper supplementation on nitrogen metabolism and volatile fatty acid production of mixed ruminal microbial growth in continuous culture flow-through fermentors. Chin. Agric. Sci. Bullet..

[107] Hernández-Sánchez D., Cervantes-Gómez D., Ramírez-Bribiesca J.E., Cobos-Peralta M., Pinto-Ruiz R., Astigarraga L., Gere J.I. (2019). The influence of copper levels on in vitro ruminal fermentation, bacterial growth and methane production. J. Sci. Food Agric..

[108] Anele U.Y., Yang W.Z., McGinn P.J., Tibbetts S.M., McAllister T.A. (2016). Ruminal in vitro gas production, dry matter digestibility, methane abatement potential, and fatty acid biohydrogenation of six species of microalgae. Can. J. Anim. Sci..

[109] Brooke Charles G., Roque Breanna M., Shaw C., Najafi N., Gonzalez M., Pfefferlen A., De Anda V., Ginsburg David W., Harden Maddelyn C., Nuzhdin Sergey V. (2020). Methane reduction potential of two pacific coast macroalgae during in vitro ruminant fermentation. Front. Mar. Sci..

[110] Makkar H.P., Tran G., Heuzé V., Giger-Reverdin S., Lessire M., Lebas F., Ankers P. (2016). Seaweeds for livestock diets: A review. Anim. Feed Sci. Technol..

[111] Machado L., Magnusson M., Paul N.A., de Nys R., Tomkins N. (2014). Effects of marine and freshwater macroalgae on in vitro total gas and methane production. PLoS ONE.

[112] Glasson C.R., Kinley R.D., de Nys R., King N., Adams S.L., Packer M.A., Magnusson M. (2022). Benefits and risks of including the bromoform containing seaweed Asparagopsis in feed for the reduction of methane production from ruminants. Algal Res..

[113] Min B.R., Parker D., Brauer D., Waldrip H., Lockard C., Hales K., Augyte S. (2021). The role of seaweed as a potential dietary supplementation for enteric methane mitigation in ruminants: Challenges and opportunities. Anim. Nut..

[114] DSM (2022). Minimizing Methane from Cattle. https://www.dsm.com/corporate/sustainability/our-purpose/minimizing-methane-from-cattle.html.

[115] Pure. https://pure.au.dk/ws/files/197951334/Notat_3NOP_BOVAER_150920.pdf.

[116] (2022). mla (Meat & Livestock Australia). https://www.mla.com.au/news-and-events/industry-news/the-feed-additive-reducing-methane-emissions-by-up-to-90/.

[117] Black J.L., Davison T.M., Box I. (2021). Methane emissions from ruminants in australia: Mitigation potential and applicability of mitigation strategies. Animals.

[118] Gill M., Smith P., Wilkinson J.M. (2010). Mitigating climate change: The role of domestic livestock. Animal.

[119] Martínez J.B.G., Pearce J.M., Throup J., Cates J., Lackner M., Denkenberger D.C. (2022). Methane single cell protein: Potential to secure a global protein supply against catastrophic food shocks. Front. Bioeng. Biotechnol..

